# Roles of BCCIP deficiency in mammary tumorigenesis

**DOI:** 10.1186/s13058-017-0907-5

**Published:** 2017-10-18

**Authors:** Roberto Droz-Rosario, Huimei Lu, Jingmei Liu, Ning-Ang Liu, Shridar Ganesan, Bing Xia, Bruce G. Haffty, Zhiyuan Shen

**Affiliations:** 1 Rutgers Cancer Institute of New Jersey, Rutgers, The State University of New Jersey, 195 Little Albany Street, New Brunswick, NJ 08903 USA; 2 Department of Radiation Oncology, Rutgers Robert Wood Johnson Medical School, Rutgers, The State University of New Jersey, New Brunswick, NJ 08903 USA; 3 Department of Medicine, Rutgers Robert Wood Johnson Medical School, Rutgers, The State University of New Jersey, New Brunswick, NJ 08903 USA

**Keywords:** Synthetic viability, BCCIP, Epidermal inclusion cyst of the breast, 53BP1, p16, Suppressor of initiation and requisite for progression (SIRP)

## Abstract

**Background:**

Dysregulated DNA repair and cell proliferation controls are essential driving forces in mammary tumorigenesis. BCCIP was originally identified as a BRCA2 and CDKN1A interacting protein that has been implicated in maintenance of genomic stability, cell cycle regulation, and microtubule dynamics. The aims of this study were to determine whether *BCCIP* deficiency contributes to mammary tumorigenesis, especially for a subset of breast cancers with 53BP1 abnormality, and to reveal the mechanistic implications of BCCIP in breast cancer interventions.

**Methods:**

We analyzed the BCCIP protein level in 470 cases of human breast cancer to determine the associations between BCCIP and 53BP1, p53, and subtypes of breast cancer. We further constructed a unique BCCIP knockdown mouse model to determine whether a partial *BCCIP* deficiency leads to spontaneous breast cancer formation.

**Results:**

We found that the BCCIP protein level is downregulated in 49% of triple-negative breast cancer and 25% of nontriple-negative breast cancer. The downregulation of BCCIP is mutually exclusive with p53 mutations but concurrent with 53BP1 loss in triple-negative breast cancer. In a K14-Cre-mediated conditional *BCCIP* knockdown mouse model, we found that BCCIP downregulation causes a formation of benign modules in the mammary glands, resembling the epidermal inclusion cyst of the breast. However, the majority of these benign lesions remain indolent, and only ~ 10% of them evolve into malignant tumors after a long latency. This tumor progression is associated with a loss of 53BP1 and p16 expression. *BCCIP* knockdown did not alter the latency of mammary tumor formation induced by conditional *Trp53* deletion.

**Conclusions:**

Our data suggest a confounding role of *BCCIP* deficiency in modulating breast cancer development by enhancing tumor initiation but hindering progression. Furthermore, secondary genetic alternations may overcome the progression suppression imposed by *BCCIP* deficiency through a synthetic viability mechanism.

**Electronic supplementary material:**

The online version of this article (doi:10.1186/s13058-017-0907-5) contains supplementary material, which is available to authorized users.

## Background

Genomic instability is a driving force of tumorigenesis, and dysregulation of caretaker genes is one of the most potent risk factors contributing to tumorigenesis [1, 2]. Genomic instability can be provoked by inaccurate repair of DNA damage, unfaithful duplication of DNA, and imprecise segregation of mitotic chromosomes [3]. As one of the leading causes of cancer fatality, breast cancer development involves dysregulation of both oncogenes and tumor suppressors. Although only ~ 20% of breast cancer has p53 mutations, triple-negative breast cancer (TNBC) has a considerably higher p53 mutation rate (50–80%) than other types of breast cancer [4–6]. Furthermore, BRCA1-associated breast cancers often harbor p53 mutations [7–11], leading to a simultaneous dysfunction of the homologous recombination (HR) pathway and the p53 network. However, a significant portion of TNBC, especially those cancers not related to *BRCA1*, do not harbor *p53* mutations [5], suggesting other molecular etiological factors may simultaneously abrogate HR and p53 functions to contribute to the subset of breast cancers that are unrelated to *BRCA1* and *p53*.

Mammalian *BCCIP* gene was initially identified as a BRCA2-interacting protein [12], and the BCCIP–BRCA2 interaction has also been reported in *Ustilago maydid* [13]. Multiple studies have suggested the critical roles of *BCCIP* in HR-dependent DNA repair, suppression of DNA replication stress, cell cycle regulation, mitosis, and ribosome biogenesis [14–22]. Mouse embryonic fibroblasts with *BCCIP* deficiency display significantly more chromatid-type aberrations [23]. A partial and transient loss of *BCCIP* was sufficient to cause medulloblastoma in mice [24]. Although human cells express two alternatively spliced BCCIP isoforms, BCCIPα and BCCIPβ [25], and BCCIPβ is the evolutionally conserved isoform from yeasts to mammals, mouse expresses only the β isoform. The human BCCIP can be coimmunoprecipitated with RAD51 [16], but only BCCIPβ can directly bind mammalian RAD51 in vitro and this interaction causes RAD51 conformational changes during HR reaction [22]. The function of BCCIP in ribosome biogenesis is also predominantly mediated by the BCCIPβ isoform [21, 26]. On the other hand, the human-specific BCCIPα isoform has gained a function in mitotic spindles and centrosomes [27]. Altogether, these studies have not only established a direct role of BCCIP in HR and genomic stability maintenance, but also suggest that BCCIP may be indispensable for cell proliferation due to its functions in cell division and ribosome biogenesis.

Of interest is that BCCIP loss has also been shown to abrogate p53 transcriptional activity [28], and BCCIP downregulation is associated with a poor prognosis in laryngeal cancer with wild-type p53 but not with mutant p53 [29]. Furthermore, the embryonic lethality in BCCIP knockdown mice cannot be rescued by codeletion of *Trp53* [30], which is distinct from the partial rescue observed with other mice deficient in other HR genes such as *Brca1* and *Palb2* [31, 32]. *BCCIP* dysfunction may therefore not only trigger HR-dependent genomic instability but also abrogate *p53* activity, which would resemble a concurrent loss of *BRCA1* and *p53*, and thus may contribute to a subset of TNBC without BRCA1 and p53 alterations. However, *BCCIP* mutation is rare in cancers based on a search of the TCGA database, *BCCIP* germline mutations are absent in breast and ovarian cancer families [33], and the *BCCIP* expression status in sporadic breast cancer tissues has not been examined. Therefore, we became interested in whether *BCCIP* deregulation can contribute to mammary tumorigenesis.

In this study, we surveyed the BCCIP protein level in breast cancer tissues, and generated a transgenic mouse model to conditionally knockdown *BCCIP* expression in the mouse mammary gland epithelium to evaluate the effects of *BCCIP* deficiency in mammary tumorigenesis. We found that *BCCIP* is downregulated in a significant portion of breast cancer, namely cancers without p53 mutation but with 53BP1 loss. *BCCIP* downregulation alone in the mouse mammary gland is sufficient to trigger the formation of benign mammary nodules but these benign lesions can hardly progress into malignant stages, supporting *BCCIP* as a suppressor for tumor initiation but a requisite for tumor progression. Furthermore, we reveal that a concurrent loss of BCCIP and 53BP1 expression may confer synthetic viability by relieving the growth arrest caused by *BCCIP* defects during the progression of benign lesions into malignancy.

## Methods

### Ethical use of animals, and generation of mouse strains with K14-Cre-mediated BCCIP dysfunction

The animal work presented in this study was approved by the Institutional Animal Care and Use Committee at Robert Wood Johnson Medical, Rutgers, The State University of New Jersey. We adhered to and followed our institutional guidelines regarding animal wellbeing. All of the models for mammary tumorigenesis described in this study were generated by crossbreeding our FVB conditional BCCIP knockdown transgenic mice (LoxPshBCCIP) as reported previously [24, 30, 34] with animals carrying the Cre recombinase gene under the control of the human keratin 14 (K14) promoter, the FVB-Tg(KRT14-Cre)8Bm/Nci strain that had been characterized previously [10]. To generate a mouse strain with *BCCIP* conditional knockdown, we crossbred FVB *LoxPshBCCIP* ^*+/+*^ females with FVB *Tg(KRT14-cre)8Bm/Nci* males to produce offspring that were *FVB:LoxPshBCCIP* ^*+/−*^ *;K14-Cre* ^*+/−*^ or *FVB*:*LoxPshBCCIP* ^*+/−*^ *;K14-Cre* ^*−/−*^, here on referred to as BCCIP-CKD *or* BCCIP-CON, respectively.

For generation of the *BCCIP-CKD;p53* ^*∆Exon2–10/∆Exon2–10*^ animals, we obtained the p53-floxed transgenic strain *B6.129-Trp53* ^*tm1Brn*^ */J* (Jackson Laboratory stock number: 008462) that has floxed exons 2–10 of p53 (from here on referred as *p53* ^*flox*^), and crossbred with our *FVB:LoxPshBCCIP* ^*+/+*^ animals. Animals confirmed homozygous for both conditional alleles were then crossed with *BCCIP-CKD* animals to generate *LoxPshBCCIP* ^*+/−*^ *;p53* ^*flox/wt*^ *;K14-Cre* ^*+/−*^ animals, here on referred to as *BCCIP-CKD;p53* ^*+/−*^. The *BCCIP-CKD;p53* ^*+/−*^ animals were then intercrossed with *BCCIP-CKD;p53* ^*+/−*^ or crossed with *LoxPshBCCIP* ^*+/+*^ *;p53* ^*flox/flox*^ animals to generate *BCCIP-CKD;p53* ^*flox/flox*^, here on referred to as *BCCIP-CKD;p53* ^*−/−*^. This compound transgenic strain was kept in a hybrid genetic background and only females were used for this study.

### Mouse genotyping by PCR

Genotype screens were performed by PCR with genomic DNA isolated from tail biopsies. The primer pair 5′-TTTTCAAGGCAATCAGGGTA and 5′-CATCACTCGTTGCATCGACC was used to amplify the 475-bp K14-Cre allele. The primer pair of 5′-TCTAGAACTAGTGGATCCGAC and 5′-AAGTTATCTCGACAAGCCTAT was used to amplify a 210-bp unrecombined U6-LoxPshBCCIP allele, the pair 5′-TCTAGAACTAGTGGATCCGAC and 5′-AGGCTTTTCTCCAAGGGATATT was used to amply a 290-bp recombined LoxPshBCCIP allele, and the pair of 5′-GGTTAAACCCAGCTTGACCA and 5′-GGAGGCAGAGACAGTTGGAG was used to amplify the 390-bp *Trp53* ^*floxExon2–10*^ and the 270-bp *Trp53* ^*wt*^ alleles respectively. The primers 5′-CACAAAAACAGGTTAAACCCA and 5′-GAAGACAGAAAAGGGGAGGG were used to yield 612-bp products for the recombined allele.

### Western blot analyses

Western blot analyses were performed with procedures, custom-made primary anti-mBCCIP antibodies (1:1000), and commercial anti β-Actin antibodies (1:2500, 4967; Cell Signaling) as described previously [27, 30].

### Histological and immunohistochemical analysis

Tissue specimens were removed surgically, washed in PBS, and fixed overnight in 10% formalin at 4 °C. After fixation, the tissue was transferred to 70% ethanol before submitting to Rutgers Cancer Institute Analytical Tissue Services for tissue processing and paraffin embedding. All formalin-fixed paraffin-embedded tissue sections were cut at 5 μM. These tissue sections were stained with hematoxylin and eosin (HE) following standard procedures. For immunohistochemical (IHC) analysis of tissue sections, antigen retrieval was carried in 0.05% citraconic anhydride (pH 7.4) [35, 36] by steaming the immersed slides in a kitchen steam cooker for 40 min after the temperature of the buffer reached 98 °C. After retrieval, slides were washed in PBS and permeabilized with 0.1% Triton X-100 in PBS for 10 min followed by quenching of endogenous peroxides with 3% hydrogen peroxide for 15 min. BSA 3% in PBS was used for blocking and dilution of primary and secondary antibodies. Incubation was carried out in a humidified chamber following standard procedures for IHC stains. Immunoreactivity was visualized with 3,3′-diaminobenzidine (DAB) (D5637; Sigma). Positive staining is visualized as a brown color precipitate that can be distinguished from the hematoxylin counterstain seen as a blue color. The evaluation of BCCIP staining was based on the same criteria as described in a previous publication [37], and tissue sections with less than 5% cells of BCCIP positive staining were recorded as BCCIP negative.

For immunofluorescent detection, fluorescein isothiocyanate (FITC) or Rhodamine conjugated secondary antibodies were used. 4′,6-Diamidino-2-phenylindole (DAPI) (H-1200; Vector Laboratories, Burlingame, CA, USA) mounting medium was used for counterstaining nuclei. The following primary antibodies were used: mBCCIP (1:100), hK14 (1:500, PRB-155P; Covance), anti-Cre (1:1000, 69050; Novagen), Ki67 (1:300, ab15580; Abcam), p16Ink4a (1:100, sc-468; Santa Cruz), and 53BP1 (1:1000, A300-272S; Bethyl). All fluorescence images were acquired by fluorescence microscopy.

### Statistical analysis

Statistical significance of tumor incidence was calculated by chi-square test, and the statistical differences of overall survival between different cohorts were analyzed by the Mantel–Cox test. *p* values and the numbers of animals in each data set are indicated in the corresponding figures and tables. Histopathological and IHC analyses were made with three tissue sections per specimen when available.

## Results

### Downregulation of BCCIP protein expression in breast cancer tissues

**Fig. 1:**
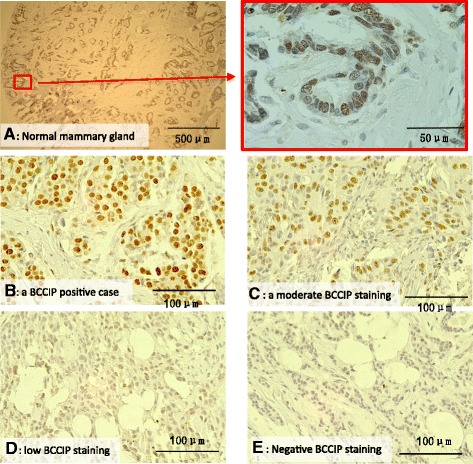
BCCIP protein expression in normal mammary glands and breast cancers. BCCIP is stained brown, and nuclei are counterstained dark blue with hematoxylin. Percentages of BCCIP nuclear staining positive cells in the tumor region were recorded and arbitrarily divided into four groups: high expression, > 40% positive cells; moderate expression, 10–40% positive cells; low expression, 5–10% positive cells; BCCIP negative, < 5% positive cells. **a** Section of normal breast tissue at low magnification with an enlarged image of the boxed area. **b** Representative case of BCCIP positively stained breast cancer. **c** Representative case with moderate BCCIP staining. **d, e** Representative cases with low or negative BCCIP staining. BCCIP BRCA2 and CDKN1A interacting protein

*BCCIP* has been described as an essential gene with significant roles in processes critical to the suppression of tumorigenesis [18–22]. Downregulation of *BCCIP* expression has been reported in brain cancer, laryngeal cancer, ovarian cancer, renal cell carcinoma, colorectal cancer, and recently among hepatocellular carcinoma [12, 25, 29, 38–40]. Despite a role of *BCCIP* in genomic stability, germline mutations in the gene are rare in breast and ovarian cancer families [33], and BCCIP expression status in sporadic breast cancer has not been examined. To further understand the association between BCCIP expression and breast cancer, we performed immunohistochemistry (IHC) staining with a BCCIP antibody on a previously described tissue microarray (TMA) composed of more than 470 core biopsies from human breast tumors [37]. We found that BCCIP protein is expressed in the normal mammary epithelium, and some tumor tissues display strong BCCIP staining. However, a considerable portion of the cases (33%) display reduced or lack of BCCIP expression (Fig. 1, Table 1). Interestingly, 49% of triple-negative breast cancer (TNBC) shows BCCIP downregulation, but only 25% of non-TNBC is BCCIP low or negative (Table 1, *p* = 3.86 × 10^–7^, χ^2^ test). Among the non-TNBC, further analysis revealed no significant association of BCCIP expression with the combination ER^+^/HER^+^ vs ER^+^/Her^–^ or ER^–^/HER^+^ vs ER^–^/HR^–^ regardless of PR status (see Additional file 1: Table S1). Furthermore, there is a strong association between BCCIP negativity and normal p53 status, or p53 mutant status with a normal level of BCCIP (Table 1, *p* = 0.0018, χ^2^ test). This is in contrast to the breast cancers of BRCA1 deficiency, which are often associated with p53 mutations [5, 8–10, 41]. We also examined BCCIP expression in 12 cases of TNBC with *BRCA1* mutations, and found that only one of the 12 cases was BCCIP negative (8%), which is significantly less frequent than in sporadic TNBC (49%, *p* = 0.0073, χ^2^ test). Overall, the observations presented in Table 1 reveal a significant downregulation of BCCIP in a subset of TNBC with normal BRCA1 and p53, indicating a potential role of BCCIP in the development of these tumors.

**Table 1:** Association of BCCIP expression with triple negativity, p53, and BRCA1 status in breast cancer tissues

	BCCIP (−)	BCCIP (+)	Total cases	*p* value
All cases	156 (33%)	317 (67%)	473	
Triple negative	66 (49%)	70 (51%)	136	3.86 × 10^–7^
Nontriple negative	80 (25%)	247 (75%)	327	
p53 wild type	133 (35%)	252 (65%)	385	0.0018
p53 mutant	12 (16%)	62 (84%)	74	
BRCA1 mutant (all triple negative)	1 (8%)	11 (92%)	12	0.0073 (compared with triple-negative tumors)

### Development of a mouse model with keratin 14-Cre-mediated conditional BCCIP knockdown

**Fig. 2:**
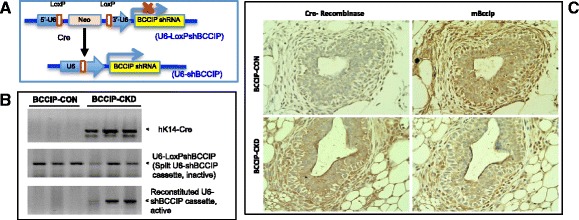
K14-Cre-mediated BCCIP conditional knockdown in the mouse mammary epithelium. **a** Strategy of Cre-mediated BCCIP knockdown. In the U6-LoxPshBCCIP construct, the U6 promoter that normally drives expression of shRNA is nonfunctional due to insertion of a LoxPNeoLoxP cassette, which can be “popped out” by expression of Cre recombinase. This allows the U6 promoter to reconstitute its activity, leading to the expression of the anti-BCCIP shRNA. **b** The hK14-Cre transgene and the LoxPshBCCIP cassette were detected in genomic DNA isolated from mammary gland tissue (top and center). PCR amplification of the recombined shBCCIP-expressing cassette was only detected in animals positive for the Cre-recombinase transgene (bottom). **c** Serial sections of formalin-fixed paraffin-embedded tissues were stained to assess BCCIP levels and corresponding Cre-recombinase expression in the mammary epithelium. Tissue sections from BCCIP-CON and BCCIP-CKD were mounted on the same slide and treated under the same conditions simultaneously. Left two panels show Cre-recombinase, right two panels show characteristic mBCCIP stain for the corresponding serial section. BCCIP BRCA2 and CDKN1A interacting protein, BCCIP-CKD *FVB:LoxPshBCCIP* ^*+/−*^ *;K14-Cre* ^*+/−*^, BCCIP-CON *FVB*:*LoxPshBCCIP* ^*+/−*^ *;K14-Cre* ^*−/−*^

To understand the role of BCCIP in mouse development, we previously generated a Cre-recombinase-based conditional BCCIP knockdown mouse model [30]. Briefly, a conditional *U6-LoxPshBCCIP* cassette was introduced into the FVB-NJ transgenic mouse line, and tissue-specific expression of Cre recombinase would restore the U6 promoter activity to allow the transcription of shRNA against BCCIP (shBCCIP) (Fig. 2a). We previously characterized the effects of BCCIP knockdown using the ubiquitous EIIa and the neuron-specific glial fibrillary acidic protein (GFAP) prompters, and BCCIP knockdown caused early embryonic lethality and neural development defects, respectively [30, 34]. Interestingly, the embryotic lethality of LoxPshBCCIP;EIIa-Cre mice was not rescued by p53 loss, but the neural development defect in the LoxPshBCCIP;GFAP-Cre mice was rescued by p53 loss [30, 34]. To define the role of BCCIP in mouse mammary tumorigenesis, we crossed the mice with the conditional *U6-LoxPshBCCIP* allele with mice that express Cre driven by the human keratin-14 promoter (hK14-Cre). The K14-driven Cre expression has been detected in stratified epithelia, including the mammary gland [10]. During early development of the rudimentary mammary tree, K14-Cre can be detected around embryonic day 15 (E15) [42]. In adult mammary glands of our *LoxPshBCCIP* ^*+/−*^ *;K14-Cre* ^*+/−*^ (BCCIP-CKD) mice, we were able to confirm that K14 is indeed expressed primarily in the epithelium (Additional file 1: Figure S1).

Females of BCCIP-CKD mice and control *LoxPshBCCIP* ^*+/−*^;*K14-Cre* ^*−/−*^ (designated BCCIP-CON) mice were monitored for any potential phenotypes. Because K14-Cre is mostly expressed in the epithelial cells, determination of the BCCIP protein level in the entire mammary gland extract is not informative on the extent of BCCIP knockdown in the basal/myoepithelial cells. We therefore used DNA genotyping of the mammalian tissues and IHC to verify the effectiveness of Cre-mediated conditional knockdown of BCCIP in the mammary glands. As presented in Fig. 2b, Cre-mediated recombination of the *U6-LoxPshBCCIP* cassette converts the split and inactive U6 promoter to a reconstituted and active U6 promoter based on genotyping on genomic DNA extracted from mammary gland tissues. After verification by IHC of the specificity of our polyclonal BCCIP antibody against mouse BCCIP in paraffin-embedded tissue sections (Additional file 1: Figure S2), we were able to conclude that the BCCIP protein level was indeed downregulated in the mammary epithelial cells when Cre is expressed (Fig. 2c). Thus, a K14-Cre-mediated conditional BCCIP knockdown model was successfully established.

### BCCIP knockdown in the mammary gland causes increased incidence of benign mammary squamous nodules

**Fig. 3:**
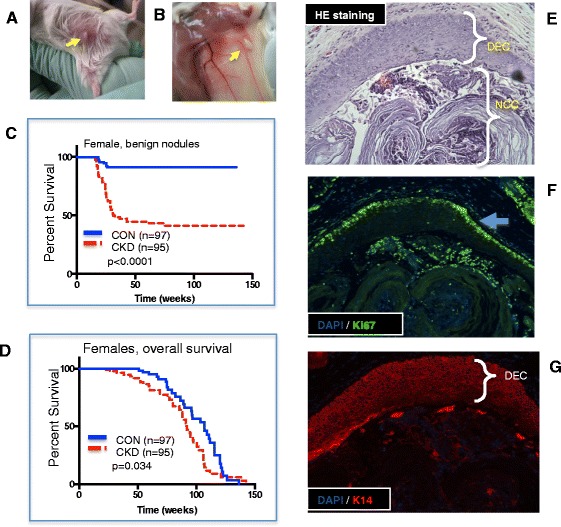
Formation of mammary nodules and overall survival of conditional *BCCIP* knockdown mice. **a, b** Gross appearance of representative mammary nodules. **c** Kaplan–Meier plots of mice surviving without formation of palpable benign nodule. **d** Kaplan–Meier plots of overall survival of BCCIP-CKD (red line) and BCCIP-Con (blue line) littermates. **e** Proliferated layers of duct epithelial cells (DEC) and the noncellular core (NCC) of the nodule. **f** Ki67 staining of a serial section from **e**. Arrow indicates the layer of Ki67 positive cells at the edge of the proliferative layers. **g** K14 staining of the serial section of **e**. Proliferated multiple layers of duct epithelial cells are K14 positive, and external layers of epithelial cells are proliferative (based on the Ki67 marker). DAPI 4′,6-diamidino-2-phenylindole, HE hematoxylin and eosin, CKD *FVB:LoxPshBCCIP* ^*+/−*^ *;K14-Cre* ^*+/−*^, CON *FVB*:*LoxPshBCCIP* ^*+/−*^ *;K14-Cre* ^*−/−*^

The BCCIP-CKD females were fertile and able to foster their litters. Analysis of mammary gland whole mounts did not reveal appreciable developmental abnormalities. Similarly, mammary ductal and acinar structures in the CKD females are unremarkable when compared to tissue sections from control animals. However, long-term observation led to the identification of discrete, palpable lesions on mammary fat pads of CKD females (Fig. 3a). The nodules were located within the mammary gland stroma, proximal to the primary duct and adjacent to the dermis, and separable from the skin layer (Fig. 3b). Often, the BCCIP-CKD females developed nodules ~ 1 mm in diameter in multiple mammary fat pads, with a median onset of about 26 weeks. As shown in Fig. 3c, the overall nodule-free survival is significantly longer in the control mice than in the CKD mice (*p* < 0.0001, Mantel–Cox test). At 6 months of age, 49 of the 154 CKD females have these nodules but only eight of the 141 control females had the same (*p* = 1.34 × 10^–8^). In addition to the increased frequency of palpable mammary nodules, the CKD females showed reduced overall life span when compared to their control littermates (Fig. 3d) (*p* = 0.034, Mantel–Cox test).

The benign lesions were typically a semisolid keratinized fibrous mass as seen in HE and Trichrome stains of paraffin-embedded sections (Fig. 3e). Normally, basal/myoepithelial cells form a single cell layer that surrounds the luminal epithelial cell layer. In the benign lesions from the CKD mice, an enlarged supra/basal K14 positive cell layer was found surrounding the nodules (Fig. 3f) and overlapping a layer of actively proliferating cells as evidenced by Ki67 positive stain (Fig. 3g). These observations suggest that downregulation of BCCIP in the mouse mammary epithelium increases the propensity for developing mammary squamous nodules.

### Malignant tumor can evolve from the benign lesions of BCCIP-deficient mice, but BCCIP deficiency decreased the incidence of de-novo breast cancers

**Fig. 4:**
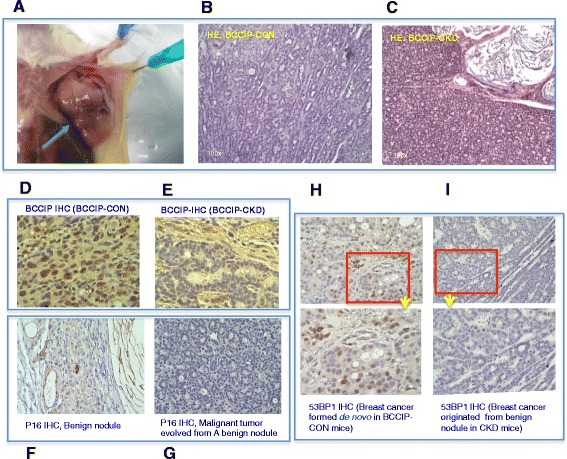
Malignant breast cancer evolved from benign nodules in *BCCIP* knockdown mice. **a** Representative mammary gland tumor evolved from a benign nodule (after 11 months of the initial detection of nodule). **b, c** HE staining of representative tumor dissected from BCCIP-CON (**b**) and BCCIP-CKD (**c**) mice. **d, e** Representative images from immunohistochemical analysis of mBCCIP status on formalin-fixed paraffin-embedded sections of mammary tumors from BCCIP-CON (**d**) and BCCIP-CKD (**e**). **f, g** IHC showing p16 (CDKN2A) in a benign lesion (**f**) and a malignant tumor (**g**) formed in BCCIP knockdown mice. **h, i** 53BP1 expression in malignant tumor evolved from benign nodules of BCCIP knockdown mice (**i**) and a de-novo tumor of wild-type mice (**h**). BCCIP BRCA2 and CDKN1A interacting protein, HE hematoxylin and eosin, IHC immunohistochemistry, CKD *FVB:LoxPshBCCIP* ^*+/−*^ *;K14-Cre* ^*+/−*^, CON *FVB*:*LoxPshBCCIP* ^*+/−*^ *;K14-Cre* ^*−/−*^

To determine whether *BCCIP* insufficiency in the mammary epithelium contributes to tumorigenesis, we continued to monitor a subset of females with benign nodules until the age of 80 weeks. While most of the benign lesions remained latent throughout the animal’s life span and did not progress further, three of the 32 palpable nodules in the CKD mice suddenly grew rapidly and the mice had to be scarified within a few days, signaling an evolution into the malignant stage, after an additional 40, 53, and 66 weeks, respectively (Fig. 4a), whereas none of the available eight nodules in the BCCIP-CON mice progressed. However, 10 de-novo malignant breast cancers developed from 10 of the 62 BCCIP-CON mice older than 50 weeks of age, but none of 54 BCCIP-CKD mice developed such tumors. It is important to emphasize that all three tumors of the BCCIP-CKD females were located with the initially detected mammary nodules; in contrast, tumors found in BCCIP-CON females all developed suddenly within a few days (thus termed de-novo tumor formation) with no prior palpable mammary growth at the site of the tumor. In addition, the malignant tumors evolved from the benign nodules of BCCIP-CKD mice displayed areas of squamous differentiation within the glandular tissue (Fig. 4c), while the de-novo tumors from the BCCIP-CON mice showed few of these features (Fig. 4b, Additional file 1: Figure S3). Compared with the de-novo tumors developed from the control mice (Fig. 4d), a reduced BCCIP protein level was found in epithelial tumor cells evolved from the benign nodules of BCCIP-CKD females (Fig. 4e). These data demonstrate that the malignant tumors formed in the BCCIP-CKD mice indeed evolved from benign nodules and originated from BCCIP-deficient cells, and they suggest that malignant cancers can develop from BCCIP knockdown mammary gland but this would likely occur in a stage-wise manner, which is in contrast to the tumors formed in wild-type mice where breast cancer formed in a de-novo fashion.

### Loss of p16Ink4a expression in malignant tumors derived from benign nodules

The increased frequency of latent benign nodules and the reduced incidence of de-novo malignant mammary tumors in the BCCIP-CKD group suggest that *BCCIP* downregulation may promote the initiation of mammary tumor formation but prohibit the de-novo tumor progression. This is consistent with a previous report suggesting that a partial and transient reduction of BCCIP is not only sufficient but also requisite for medulloblastoma formation, likely due to its concurrent roles in the maintenance of genomic integrity and supporting cell proliferation [24]. Because the majority of the benign lesions on BCCIP-CKD females did not further progress to malignant neoplasms and there was an extended latency (at least 9 additional months) between benign nodule formation and malignant transformation for those that did, we wondered about the molecular changes that may be associated with the transition of the benign lesions into the malignant form despite the persistent BCCIP downregulation. Since p16Ink4a has been used as a marker of senescent cells and loss of its expression is associated with multiple malignant neoplasms including breast cancer [43, 44], we compared the expression of p16Ink4a protein in the benign nodules and the malignant tumors that evolved from them. As shown in Fig. 4f, g, while there was strong expression of p16 protein in the benign lesions, the expression was lost in the malignant tumors, indicating that loss of p16 expression maybe required for the benign lesions to escape a quiescent state and evolve into malignant tumors.

### Decreased expression of 53BP1 in BCCIP-deficient mammary tumors

Because *BCCIP* deficiency would impair homologous recombination (HR) and hinder cell proliferation, it was unknown how some of the benign nodules (although a relatively rare event at a frequency of 3/32) evolved into malignant tumor while BCCIP remained downregulated (Fig. 4e). It is known that a synthetic viability conferred by 53BP1 loss may play a role in the development of BRCA1-deficient breast cancer [7, 37, 45–50]. This is because 53BP1 loss can partially restore HR activity in the absence of BRCA1 [46, 51, 52]. Therefore, we measured 53BP1 levels in malignant tumors developed from the CKD mice. As shown in Fig. 4h, i, regions with epithelial cell morphology in the tumors displayed negative staining for 53BP1, whereas stromal cells and other cells of nonepithelial origin on the same slide reacted with 53BP1 antibody, suggesting a reduced 53BP1 expression in the tumor cells. Remarkably, loss of 53BP1 staining was found in all three cases of malignant tumor evolved from the benign nodules of the CKD mice, while all three tumors with normal BCCIP tested showed normal 53BP1 expression. This suggests that loss of 53BP1 expression may be an accompanying molecular change and may be required for the BCCIP-deficient benign lesions to evolve into malignant tumors, which would resemble the relationship between BRCA1 and 53BP1 in tumor development [7, 45–47].

In light of these observations, we further surveyed the relationship between BCCIP expression and 53BP1 expression in the same set of human breast cancers as presented in Table 1 and a previous report [37]. Interestingly, we found a strong correlation between BCCIP negativity and 53BP1 negativity in TNBC, but not in non-TNBC (Table 2). This finding is consistent with the notion that loss of 53BP1 is associated with BCCIP deficiency in breast cancer, and indicates a potential synthetic viability relationship between BCCIP and 53BP1 deficiencies.

**Table 2:** Association of BCCIP with 53BP1 in triple-negative and nontriple-negative breast cancers

Breast cancer	BCCIP (−)	BCCIP (+)	Total cases	*p* value
Triple negative				
53BP1 (−)	40	15	55 (43.0%)	3.63 × 10^–7^
53BP1 (+)	20	53	73 (57.0%)	
Subtotal	60 (46.9%)	68 (53.1%)	128	
Nontriple negative				
53BP1 (−)	2	4	6 (1.9%)	0.58
53BP1 (+)	71	231	302 (98.1%)	
Subtotal	73 (23.7%)	235 (76.3)	308	

*BCCIP* BRCA2 and CDKN1A interacting protein

### BCCIP deficiency does not significantly synergize with p53 loss to promote tumorigenesis

Previously, we noted that BCCIP is required for wild-type p53 activity [15, 28]. Loss of BCCIP expression in laryngeal cancers was associated with worse prognosis after radiation therapy but this was only true for tumors with wild-type p53 [29]. Consistent with these reports, BCCIP negativity was found to be associated with breast cancers of wild-type p53 (Table 1). These observations imply that BCCIP’s role in breast cancer may be independent of p53 status. However, p53 loss was able to rescue the brain development defect caused by BCCIP knockdown and to promote medulloblastoma [24, 34], suggesting that p53 loss can contribute to BCCIP deficiency-induced tumor formation, at least in the context of medulloblastoma. Therefore, we asked whether there could be synergy between BCCIP deficiency and p53 loss for mammary tumorigenesis.

**Fig. 5:**
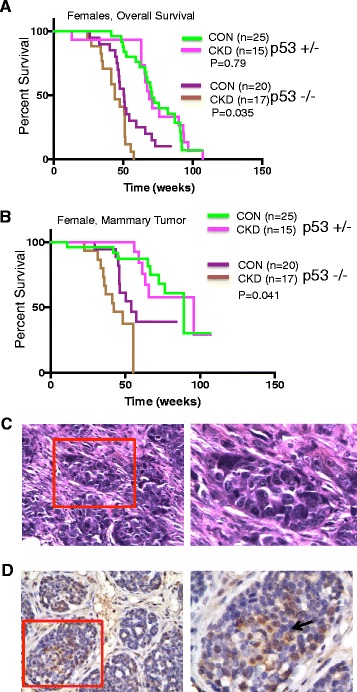
Mammary tumor formation in p53^–/–^ mice. **a** Overall survival of BCCIP-CON and BCCIP-CKD mice with concurrent homozygous and hemizygous p53 deletions. **b** Mammary tumor-free survival of BCCIP-CON and BCCIP-CKD mice with concurrent homozygous and hemizygous p53 deletions. **c** HE stains of formalin-fixed paraffin-embedded sections from mammary tumors on BCCIP-CKD;p53^–/–^ females. Right panel shows a higher magnification from the highlighted area to show squamous cells within the tumors (yellow arrows). **d** IHC of breast cancer derived from BCCIP-CKD and p53^–/–^ mice. Right panel shows a higher magnification of clusters of BCCIP positive cells (black arrows) within the mostly BCCIP negative tissue in the tumor specimens. CKD *FVB:LoxPshBCCIP* ^*+/−*^ *;K14-Cre* ^*+/−*^, CON *FVB*:*LoxPshBCCIP* ^*+/−*^ *;K14-Cre* ^*−/−*^

We crossed our BCCIP-CKD (*LoxPshBCCIP;K14-Cre*) mice with mice carrying floxed Trp53 alleles (*Trp53* ^flox/flox^), producing four different genotypes: LoxPshBCCIP^+^:*Trp53* ^flox/flox^, LoxPshBCCIP^+^:*Trp53* ^flox/wt^, *Trp53* ^flox/flox^, and *Trp53* ^flox/wt^ (all with K14-Cre). We found that deletion of either one or both copies of *Trp53* resulted in earlier tumors compared with mice with wild-type *Trp53* (Fig. 5a with Fig. 3d). However, downregulation of BCCIP in these mice did not synergistically promote tumor-associated death (Fig. 5a). Many female mice with homozygous or hemizygous loss of p53, regardless of their BCCIP status, developed mammary tumors and skin cancers (Additional file 1: Table S1). It should be emphasized that all tumors which developed from the *Trp53* ^flox/flox^ or *Trp53* ^flox/wt^ mice were “de novo” and did not evolve from the aforementioned benign nodules. Although coknockdown of BCCIP slightly accelerated mammary tumor formation in *Trp53* ^flox/flox^ mice (Fig. 5b) (*p* = 0.041), the overall frequencies of breast cancer tumor incidence are similar between BCCIP-CON and BCCIP-CKD mice (Additional file 1: Table S2). Among the limited number of male mice with *Trp53* deletion, skin cancer and skin ulcer were often observed in both the BCCIP-CON and the BCCIP-CKD cohorts (Additional file 1: Table S3). This is consistent with the fact that K14 promoter is also active in the skin epithelial cells [53].

As seen in HE-stained sections, p53-deficient mammary tumors contained regions of squamous differentiation within glandular tissue and other histological differentiation patterns (Fig. 5c). Different from the BCCIP-CKD tumors that evolved from the benign nodule, there was no consistent BCCIP reduction in the tumors that formed in the *LoxPshBCCIP* ^*+*^;*Trp53* ^flox/flox^ or *LoxPshBCCIP* ^+^;*Trp53* ^flox/wt^ mice, as pockets of BCCIP positive cells were observed in the tumors (Fig. 5d). These observations indicate that loss of tumor suppressor activities of p53 by itself was sufficient to initiate neoplastic transformation, consistent with reports of other mouse models utilizing K14-Cre [9, 10, 54]. Although unexpected, our observations suggest a possibility of synergism between BCCIP deficiency and p53 loss in mouse mammary tumor development. This is different from their synergistic effect in the rapid medulloblastoma development when BCCIP and p53 were coablated in the GFAP-Cre mouse model [24], but consistent with the observations made in human breast cancer (Table 1).

## Discussion

In this study, we found that the BCCIP protein level is downregulated in 33% of 473 cases of breast cancers (49% among triple-negative vs 25% among non-TNBC, *p* = 3.86 × 10^–7^) and BCCIP loss tends to be exclusive with p53 mutations but concurrent with 53BP1 loss in triple-negative cases (Tables 1 and 2). K14-Cre-mediated BCCIP knockdown in the mammary gland led to increased benign nodule formation, but only ~ 10% of these lesions eventually evolved into malignant status and this was accompanied by loss of 53BP1 and p16 expression. These observations suggest that BCCIP deficiency can induce tumor initiation but may hinder the subsequent progression, unless additional events occur to allow the malignant transformation. This reinforces the concept that BCCIP serves as a unique type of tumor suppressor previously called SIRP: a suppressor of initiation but a requisite for progression [24].

The benign lesions in BCCIP-CKD mice are well-circumscribed solid masses located in the mammary stroma. These nodules were detected in the mammary fat pads, adjacent to the primary ducts and proximal to the nipples (Fig. 3). Although the precise origin of these squamous nodules is not clear, the characteristic lamellar keratin resembles that of epidermal inclusion cyst (EIC) of the breast [55]. EICs are typical of cystic lesions with lamellar keratin, oval in shape and with a multiple epithelial cell layer surrounding the deposited material [56, 57]. There is no consensus on the origin of these benign lesions in the breast, but several theories have been put forth, including the possibility of metaplastic squamous differentiation of amplified epithelial duct cells [56, 57]. It is generally appreciated that these benign tumors can remain quiescent for many years before they undergo transformation to malignant tumors [57]. Based on an analysis of 82 patients with EICs in the breast, 10 cases (~12%) were transformed into squamous cell carcinoma in the breast [55, 57–59].

A question of interest is why *BCCIP* knockdown causes the quiescent status of the benign lesions. In this study, we found that the p16 is expressed in the epithelial cells of benign nodules, which is a marker of cellular senescence and a known barrier for tumor progression [43, 60–62]. Interestingly, the transition to malignance is associated with a loss of p16 expression (Fig. 4f, g). It is possible that the partial loss of BCCIP caused sufficient genome instability or other disturbances in the affected mammary epithelial cells to initiate abnormal proliferation, but also triggered senescence in response to excessive spontaneous DNA damage, which may lock the initially proliferative benign lesion into a quiescent stage and cause the majority of the EIC-like nodules to stay benign for the entire lifespan unless a subsequent, stochastic genetic event causes the loss of p16 expression.

Although the majority of benign lesions remained quiescent for a long time, three of these 32 lesions indeed evolved into malignant tumors after more than 9 months despite having reduced (but not complete loss of) BCCIP expression. Interestingly, we found that all three malignant tumors evolved from benign lesions had lost the expression of 53BP1 protein, and this association between 53BP1 negativity and BCCIP downregulation was also found in human TNBC (Table 2). Thus, it is possible that loss of 53BP1 may have overcome the partial BCCIP loss to promote progression of the benign nodules, a possibility that needs to be further tested with new mouse models. Data in Table 1 also suggest that a subset of TNBC may not be in association with BRCA1 and p53 dysfunction, a concept that has also been proposed [63]. Our data further raise the possibility that concurrent BCCIP and 53BP1 loss may be accountable for the subset of TNBC that are not associated with concurrent defects of BRCA1 and p53, a scenario worthy of further investigation.

In a previous study, GFAP-Cre-driven, shRNA-mediated partial loss of BCCIP in brain tissue was sufficient to cause neurodegeneration and microcephaly accompanied by p53 activation [34]. However, microcephaly and neurodegeneration were rescued by *Trp53* deletion and this led to tumor development [24], suggesting synergy between BCCIP loss and p53 loss in the development of medulloblastoma. In this study, when the K14-Cre-driven *Bccip*-CKD mice were crossed with the same conditional *Trp53* mice as used previously [24], we did not observe any synergy in tumor formation or overall survival. The lack of synergies between p53 loss and BCCIP loss in this mouse model is in agreement with the observation of human breast cancer (Table 1). We also speculate that since p53 deficiency alone caused tumor development with a relatively short latency (when compared to BCCIP deficiency) it may have masked the opportunity for cells with concurrent p53 and BCCIP loss to develop breast cancer.

BCCIP protein was detectable in tumor formed in BCCIP-CKD;p53^−/−^ mice (Fig. 5d), suggesting that the tumors may not be derived from BCCIP-deficient cells, when *Trp53* was deleted. Loss of tumor suppressor activities of p53 itself in the progenitor cells of the mammary epithelium is sufficient to initiate a rapid neoplastic transformation [10, 54]. The same is true for skin cancers. This tumor development is so quick and penetrant that it may precede and completely mask tumor development driven by BCCIP deficiency, which is likely a slower and longer process. Thus, additional mouse models are needed to test the epispastic interactions between p53 and BCCIP in breast cancer.

**Fig. 6:**
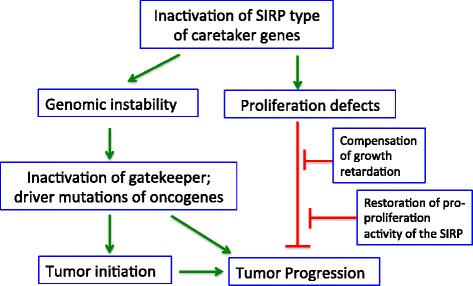
A two-step model for a SIRP (suppressor of initiation but a requisite for progression) type of tumor suppressor to contribute to tumorigenesis. On one hand, a defect of SIRP tumor suppressor triggers genomic instability and tumor initiation. On the other, the same defect impairs cell proliferation and thus hinders further progression of the tumors. However, restoration of the SIRP activity in a later stage, or a secondary genetic change that compensates the SIRP defects, may confer synthetic viability to allow the tumor to overcome the growth-retardation barrier

Previously, we reported that a partial and transient loss of BCCIP was sufficient to initiate medulloblastoma formation but a restoration of BCCIP level is required for the progression of medulloblastoma in mice [24]. This work led us to propose BCCIP as a unique type of caretaker called SIRP: suppressor for initiation but a requisite for progression [24]. In keeping with the notion of the dual role of BCCIP in tumorigenesis, we observed that a significant number of the BCCIP-CKD mice developed benign lesions in the mammary gland, but the majority of these lesions remained quiescent. Nonetheless, a small portion of the benign lesions evolved into mammary tumors after a long latency. Thus, the question is how the quiescent benign lesions can overcome the BCCIP deficiency to evolve into malignancy. In the case of medulloblastoma that developed in BCCIP-deficient mice, the tumors arose from cells in which the shBCCIP-expressing cassette had been deleted and BCCIP expression restored [24]. However, in the breast cancers that evolved from the benign lesions of BCCIP-CKD mice, BCCIP levels remained reduced, suggesting that an alternative genetic change(s) may have occurred to bypass the dependence on BCCIP for tumor progression. Interestingly, we found that all three cases had lost 53BP1 expression (Fig. 4h, i), which is consistent with the observed codownregulation of BCCIP and 53BP1 in human breast cancer (Table 2). The long latency and loss of 53BP1 and p16 indicate that additional mutagenic events can compensate for BCCIP dysfunction and achieve malignant progression. Thus, we propose a model in which at least two alternative mechanisms can enable the SIRP-deficiency-initiated tumors to overcome the proliferation barrier to progress: either by directly restoring the expression of BCCIP or by other changes to confer a synthetic viability with BCCIP loss (Fig. 6).

Another critical question is why a partial BCCIP deficiency promotes the initiation of tumorigenesis. This may be explained by the multiple functions of BCCIP in the maintenance of genomic integrity. In earlier studies, we showed that only partial BCCIP knockdown was sufficient to cause accumulation of spontaneous DNA damage and replication stress, chromosome aneuploidy, and cell cycle deregulation [13–20, 28, 30]. It is therefore plausible that a hypomorphic BCCIP function is sufficient to elicit genomic instability. As a matter of fact, we showed previously that GFAP-Cre-mediated 50–70% downregulation of BCCIP in mouse brain was sufficient to trigger medulloblastoma. Importantly, the resulting tumors invariably had large deletions or rearrangements of the *Patch1* gatekeeper gene [24], which is consistent with the notion that partial BCCIP deficiency triggered genomic instability through a defect in DNA double-strand break repair or replication stress [24]. The next question is why BCCIP defect may hinder tumor progression and recovery of BCCIP function is a requisite for tumor progression. We propose that at least three functions of BCCIP are indispensable for cell proliferation. In addition to the function in DNA replication as summarized, BCCIPβ was reported to function in ribosome biogenesis [21, 26], and the human BCCIPα (and mouse BCCIP) is required for mitosis [27]. Because of these critical functions of BCCIP in cell growth and division, it is understandable that a complete or severe loss of BCCIP may be detrimental for cell proliferation and thus BCCIP becomes a requisite for tumor progression.

## Conclusions

Our study shows that a significant fraction of human breast cancers has reduced BCCIP expression. Although a partial loss of BCCIP in murine mammary basal epithelial cells is able to generate benign squamous nodules, most of the lesions remained growth-arrested. Nonetheless, some of the benign lesion evolved into malignant tumor after a long latency, and this was associated with the loss of 53BP1 expression. Our study supports the notion that BCCIP is a unique caretaker suppressor of tumor initiation but a requisite for tumor progression, and BCCIP dysfunction has important roles in modulating mammary tumorigenesis.

## Additional file

Additional file 1: Table S1. Presenting BCCIP protein expression in subtypes of non-TNBC, **Table S2** presenting tumor incidence and average age (weeks) of onset with K14-Cre-mediated p53 deletion, **Table S3** presenting skin cancer incidence and average onset age (weeks) with K14-Cre-mediated p53 deletion, Figure S1 showing hK14 is expressed and detected in myoepithelial/suprabasal cells in the mammary gland of adult FVB-NJ mice, Figure S2 showing validation of mouse BCCIP antibody for IHC staining with paraffin-embedded tissues, and Figure S3 showing histology of two pairs of additional malignant tumor from BCCIP-CKD and BCCIP-CON mice. (PDF 5014 kb)

## Abbreviations

BCCIP: BRCA2 and CDKN1A interacting protein
CKD: Conditional knockdown
EIC: Epidermal inclusion cyst
HR: Homologous recombination
TNBC: Triple-negative breast cancer
