# Supplementary material for: Roles of BCCIP deficiency in mammary tumorigenesis
Source: Breast Cancer Res. 2017 Oct 18;19:115. doi: 10.1186/s13058-017-0907-5 (PMC5648485; doi:10.1186/s13058-017-0907-5)
Supplement: Additional file 1: Table S1. — Presenting BCCIP protein expression in subtypes of non-TNBC, Table S2 presenting tumor incidence and average age (weeks) of onset with K14-Cre-mediated p53 deletion, Table S3 presenting skin cancer incidence and average onset age (weeks) with K14-Cre-mediated p53 deletion, Figure S1 showing hK14 is expressed and detected in myoepithelial/suprabasal cells in the mammary gland of adult FVB-NJ mice, Figure S2 showing validation of mouse BCCIP antibody for IHC staining with paraffin-embedded tissues, and Figure S3 showing histology of two pairs of additional malignant tumor from BCCIP-CKD and BCCIP-CON mice. (PDF 5014 kb) [file 13058_2017_907_MOESM1_ESM.pdf]

Table S1, BCCIP protein expression in subtypes of non-triple negative breast cancers

	BCCIP (-)	BCCIP (+)	Total cases	
Total Non-TNBC	70 (22.4%)	243 (77.6%)	313	
ER-/Her2-	15 (40.5%)	22 (59.5%)	37	P=0.4233
ER-/Her2+	10 (31.3)	22 (68.7)	32	
Total	25 (30.4%)	44 (69.6%)	69	
ER+/Her2-	39 (19.9)	157 (80.1%)	196	P=0.8847
ER+/Her2+	10 (20.8%)	38 (79.2%)	48	
Total	49 (20.1%)	195 (79.9%)	244	
ER-	21 (30.4%)	48 (69.6%)	69	P=0.0684
ER+	49 (20.1%)	195 (79.9%)	244	
Total	70 (22.4%)	243 (77.6%)	313	
Her2-	54 (23.2%)	179	233	P=0.7404
Her2+	20 (25%)	60 (75%)	80	
Total	74 (23.6%)	239 (76.4)	313	
Non-TNBC DCIS	13 (32.5%)	27 (67.5%)	40	P= 0.1580
Non-TNBC non NCIS	61 (22.3%)	212 (77.7%)	273	
Total	74 (23.6%)	239 (76.4%)	313	
DCIS-TNBC	5 (38.5)	8 (61.5%)	13	P=0.6933
DCIS- Non-TNBC	13 (32.5%)	27 (67.5%)	40	
Total	18 (34.0%)	35 (66.0%)	53	

Table S2. Tumor incidence and average age (weeks) of onset with k14-Cre mediated p53 deletion.

P53 state	BCCIP status	Total # of mice	Mice with mammary gland tumors	Mice with skin tumors	Total # of mice with tumors *	P-value between total tumor incidence of CON and CKD
p53-/-	BCCIP-CON	20	9 (46.5 wk)	11 (45.1 wk)	16 (45.7 wk)	P=0.2106
	BCCIP-CKD	17	10 (38.3 wk)	11 (40.8 wk)	16 (38.1 wk)	
p53+/-	BCCIP-CON	25	9 (61.8 wk)	16 (58.6 wk)	16 (58.6 wk)	P=0.1201
	BCCIP-CKD	15	6 (66.9 wk)	8 (71.2 wk)	13 (70.1 wk)	

* Some of the mice developed multiple tumors including mammary, skin, and occasionally other tumor type, but they were counted as one mouse with tumor.

Table S3. Skin cancer incidence and average onset age (in weeks) with k14-Cre mediated p53 deletion.

		Sex	Total # of mice	Mice with skin tumors	P-value
p53 -/-	BCCIP-CON	Female	20	11 (45.1 weeks)	P=0.024
		Male	18	11 (36.2 weeks)	
		Total	38	22 (40.9 weeks)	
	BCCIP-CKD	Female	17	11 (40.8 weeks)	
		Male	9	6 (43.5 weeks)	
		Total	26	22 (38.4 weeks)	
p53 +/-	BCCIP-CON	Female	25	16 (58.6 weeks)	P= 0.46
		Male	15	6 (50.7 weeks)	
		Total	40	22 (56.5 weeks)	
	BCCIP-CKD	Female	15	8 (71.2 weeks)	
		Male	5	5 (62.0 weeks)	
		Total	20	13 (67.6 weeks)	

Figure S1

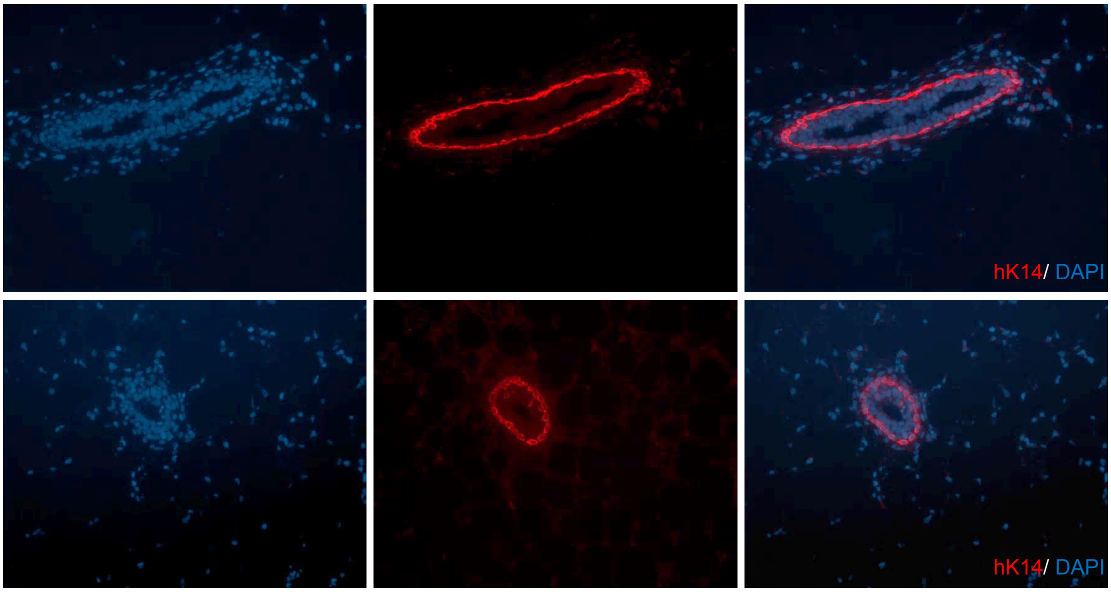

Figure S1 : hK14 is expressed and detected in myoepithelial/ suprabasal cells in the mammary gland of adult FVB-NJ mice. Paraffin embedded tissue sections were processed and incubated with hK14 antibody. Top panel shows a representative image of a mammary duct where a single layer of K14 (red) reactive cells that surrounds a layer of luminal cells visible with DAPI. The bottom panels show a cross section view of a mammary duct where the concentric layers of epithelial cells is appreciated and the k14 positive cells locate to the most outer layer in the structure.

Figure S2

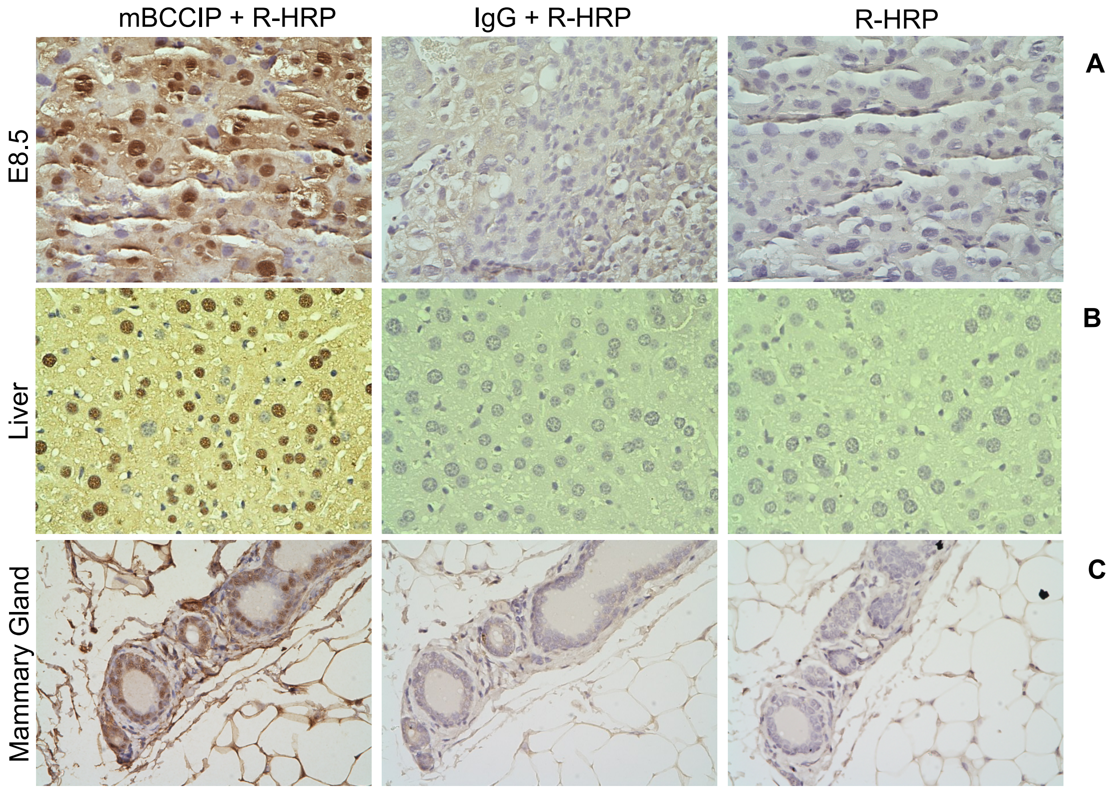

Figure S2: Validation of mouse BCCIP antibody for IHC staining with paraffin embedded tissues. Paraffin embedded tissue sections from: mouse embryos, mouse liver, and mouse mammary glands were subjected to heat induced antigen retrieval and then incubated with *mBCCIP* antibody (left panels), non-immune immunoglobins (center panels), or with sera used as diluent (right panels). mBCCIP antibody reacts with specificity in mouse tissues and locates primarily to the nuclear compartment in liver but with some reactivity in cytoplasm of embryonic and mammary gland tissues. R-HRP: Rhodamine conjugated HRP.

Figure S3

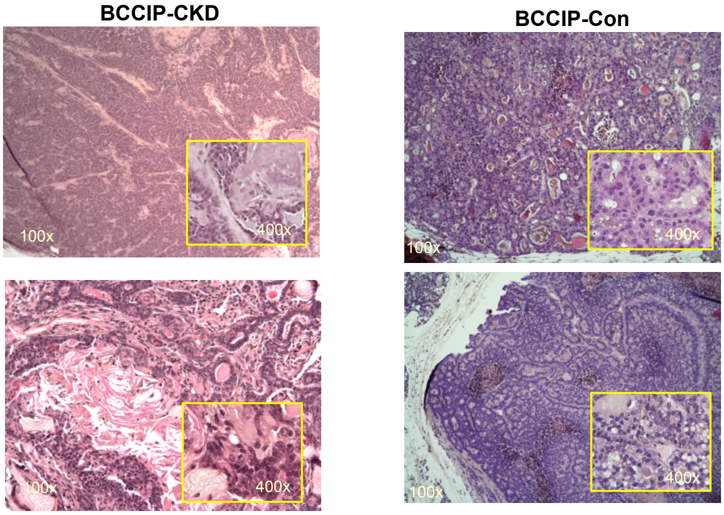

Figure S3, Histology of 2 pairs of additional malignant tumor from BCCIP-CKD and BCCIP-Con mice.
